# Deep Learning for Analysis of Bone Marrow Adiposity: Breakthroughs from Recent Large-Scale Analyses in the UK Biobank

**DOI:** 10.1007/s11914-026-00953-6

**Published:** 2026-03-10

**Authors:** Wei Xu, Chengjia Wang, William P Cawthorn

**Affiliations:** 1 Centre for Global Health, Usher Institute, University of Edinburgh, EH8 9AG Edinburgh, UK; 2 Institute for Neuroscience and Cardiovascular Research, University of Edinburgh, Edinburgh BioQuarter, 47 Little France Crescent, Edinburgh, EH16 4TJ UK; 3 Edinburgh Imaging, University of Edinburgh, The Queen’s Medical Research Institute, Edinburgh BioQuarter, 47 Little France Crescent, Edinburgh, EH16 4TJ UK; 4 School of Mathematics and Computer Sciences, Heriot-Watt University, Edinburgh, EH14 1AS UK

**Keywords:** Bone marrow adiposity, Bone marrow adipose tissue, Deep learning, GWAS, PheWAS, UK biobank

## Abstract

**Purpose of Review:**

Bone marrow adipose tissue (BMAT) is a significant fat depot with distinct skeletal, haematological and metabolic roles. It increases with ageing, osteoporosis, metabolic disease, and cancer, and is emerging as a biomarker for fracture risk. Quantification of bone marrow adiposity (BMA) has relied on MRI, proton spectroscopy, computed tomography, or histology, but large-scale studies have been limited by requiring labour-intensive analysis. Deep learning (DL) now enables scalable, automated BMA measurement, with greatest progress in MRI. This review highlights recent advances.

**Recent Findings:**

DL models, especially U-Nets, have been applied to UK Biobank MRI data, allowing site-specific BM fat fraction (BMFF) measurement or calvarial BMA estimation in tens of thousands of participants. These breakthroughs have revealed robust associations between BMA and age, sex, ethnicity, bone mineral density, adiposity, and metabolic traits, while uncovering site-specific patterns. Genome-wide association studies of BMFF and calvarial BMA have defined their genetic architecture, identifying hundreds of loci enriched for pathways in oestrogen signalling, adipogenesis, and skeletal remodelling. Phenome-wide association studies demonstrate links between altered BMFF and osteoporosis, fracture, type 2 diabetes, cardiovascular disease, and diverse other conditions, with Mendelian randomisation providing the first causal evidence that increased femoral BMFF contributes to osteoporosis. Despite successes, challenges remain, including extending analyses to non-European ancestries and validating DL pipelines in clinical settings.

**Summary:**

Collectively, DL-enabled BMA quantification has established BMAT as a clinically relevant, genetically tractable fat depot and provides new opportunities for mechanistic insight, risk prediction, and therapeutic targeting in musculoskeletal, metabolic, and other diseases.

## Introduction

The bone marrow (BM) is a site of substantial fat storage, accounting for ~ 10% of total fat mass in healthy lean humans [1]. This BM adipose tissue (BMAT) further increases with ageing and numerous clinical conditions, including osteoporosis, metabolic syndrome, and treatment responses in cancer and haematological disease. Smaller-scale preclinical and clinical studies have suggested distinct roles of BMAT in regulating skeletal remodelling, haematopoiesis, and local and systemic metabolism, including through secretion of paracrine and endocrine effectors [1]. Increased BM adiposity (BMA) has also attracted interest as a new biomarker for fracture risk [2, 3, 4, 5]. Thus, BMAT is increasingly recognised as a critical component of skeletal biology and systemic metabolism that may directly influence health and disease.

BMA can be quantified through several imaging modalities, including magnetic resonance imaging (MRI) with or without proton spectroscopy (^1^H-MRS); computed tomography (CT), including dual-energy CT and high-resolution peripheral quantitative CT (HR-pQCT); and histology, including quantitative histomorphometry [6, 7, 8, 9]. However, using these methods for large-scale BMAT assessment in humans has been limited by labour-intensive manual image segmentation, small sample sizes, and/or highly site-specific protocols. This has limited our understanding of the pathophysiological functions of BMAT and the clinical relevance of altered BMA.

The advent of deep learning (DL) methods is now transforming this landscape, enabling scalable, automated, and reproducible quantification across large datasets. This review addresses the state of the art in DL for measuring BMA, beginning with a modality-specific overview. We then focus on DL applied to MRI data, providing an in-depth exploration of recent landmark studies reporting large-scale analyses in the UK Biobank (UKB) [10, 11, 12, 13, 14, 15, 16]. We discuss how these studies have advanced understanding of BMAT formation and function, highlighting key breakthroughs. Finally, we outline future challenges and opportunities, including for clinical translation, in the rapidly growing field of BMA research.

## DL for BMA Quantification Across Modalities

### Differences in Methods for BMA Quantification

The prevalence of DL for BMA analysis differs widely across modalities. Most progress has been made for MRI which, together with ^1^H-MRS, is considered the gold standard for non-invasive BMAT quantification [6, 8]. Using multi-echo Dixon or IDEAL sequences, MRI enables the separation of water and fat signals to compute the voxelwise BM fat fraction (BMFF) or the proton density fat fraction (PDFF). In contrast, ¹H-MRS offers a localised biochemical fingerprint of BM lipid fraction and composition, including saturation and unsaturation levels [6, 8].

Unlike MRI, CT-based BMA measurements rely on differences in the physical density of bone and BM components, with BMAT being less dense than red BM or bone [7, 17]. Single-energy CT can thereby identify BMAT-rich vs. BMAT-deficient BM, but it does not allow precise quantification of BMFF or BMAT volume. In contrast, dual-energy CT provides more-accurate BMFF estimates that show close correlation to those from MRI or ¹H-MRS [7, 18]. Similar advances have recently been reported for BMFF estimation from HR-pQCT data, which, unlike conventional CT, allows BM voxels to be analysed separately to those for trabecular bone [9].

Histological BMA measurement differs to the above methods in that it analyses ex vivo samples rather than whole tissues *in vivo.* One benefit is that histology can precisely assess BM adipocyte sizes and spatial relationships with other skeletal cell types, providing distinct mechanistic insights into BMAT formation and function.

### DL for Segmentation of Bone and BM

Despite these differences, a common feature of MRI, CT-based methods, and histology is the need to define regions of interest (ROIs) for further analysis, whether for BMFF/PDFF calculations, analyses of BM density, or adipocyte histomorphometry. Traditionally, these ROIs require manual segmentation from the source imaging data, often with a second manual operator to validate the results. This time-consuming analysis limits throughput, which has restricted the scope of BMA studies. DL methods can overcome these limitations by allowing rapid, automatic ROI detection and segmentation for specific skeletal compartments. Popular DL models include 3D convolutional neural networks (CNNs), especially those using the U-Net architecture [19]. The U-Net symmetric encoder-decoder structure and “skip connections” allow it to preserve high-resolution spatial details while also incorporating broader contextual information from the image. Thus, U-Net is now a widely recognised standard in biomedical image segmentation due to its ability to perform highly accurate segmentation, even with limited training data [19]. While frontier models such as Transformers [20], diffusion models [21], and foundation models [22] are advancing medical imaging, convolutional architectures currently remain advantageous for BMA analysis due to their superior generalisability when training data are scarce.

DL methods are increasingly widespread for bone segmentation from CT data [23, 24, 25, 26] and for histological quantification [27, 28, 29]; however, only a handful of such DL models have been developed for BMA measurement. For example, MarrowQuant 2.0 can accurately segment adipocytes from BM biopsies, thereby quantifying adipocyte numbers, size distributions, and total adipose area for comprehensive histological BMA analysis [30]. More recently, Imani et al. reported a DL pipeline for CT scans of the proximal hip, allowing automatic segmentation of several musculoskeletal tissues, including BMAT [31]. Yet despite these advances, DL pipelines for measuring BMA from CT or histology remain rare.

### DL Methods for ^1^H-MRS

The same is true for ^1^H-MRS, despite its position as a gold standard for quantifying BMA. This is largely because most radiological DL models have been developed for image segmentation, whereas MRS requires spectroscopic quantification. However, DL has several applications for MRS that could benefit BMA analysis [32]. For example, while MRS is highly specific, it is limited by voxel size and susceptibility to artefacts; hence, DL models have been developed for voxel-wise quality control (QC), including identification and filtering out of poor-quality spectra and artefact removal [32]. DL models could also be trained to automatically identify and quantify fat and water peaks from complex MRS spectra. This could improve speed and consistency compared to manual fitting, even under low signal-to-noise ratios [33, 34].

Despite this potential, most BM ^1^H-MRS studies still rely on manual QC and traditional fitting, highlighting a notable research gap. Integrating DL could potentially automate and accelerate BMA analyses from these complex spectra, including reducing inter-operator variability and helping to standardise analyses for studies conducted across multiple research sites.

### DL for MRI-Based BMA Quantification

Far more progress has been made in DL for MRI-based BMA analyses: U-Net models have been used to segment BM from individual vertebrae, groups of vertebrae, the pelvis, and different locations within the leg, including the femoral head, total hip, combined proximal femur (head + total hip), femoral diaphysis, and the knee [10, 11, 12, 14, 15, 16, 35, 36, 37, 38]. Some of these studies involved only tens to hundreds of people [35, 36,37]; however, seven recent studies report DL-based BMA analysis from tens of thousands of participants in the UK Biobank (UKB) imaging study [10, 11, 12, 13, 14, 15, 16]. Below we provide an overview of these studies, including their different DL pipelines, identification of anthropometric associates, genetic architecture, and clinical implications of BMA.

## Summary of DL for Large-Scale Analysis of BMA in the UK Biobank

Two of the UKB BMA studies, from our group, are based on the lightweight attention-based 3D U-Net that we reported in 2024 [38]. Our model, which was trained and validated on a subset of UKB dual-echo MRI data, segments ROIs within the spine, femoral head, total hip, and femoral diaphysis (Table 1); such multi-site analyses are critical because BMAT has site-specific characteristics [1]. By applying this DL pipeline to the UKB dual-echo MRI data, we have measured BMFF at these four sites in over 48,000 UKB participants [10, 11] (Table 1). This has allowed identification of anthropometric associations, and, through genome-wide association studies (GWAS) and phenome-wide association studies, comprehensive identification of the genetic variants and diseases associated with altered BMFF at each site. As discussed below, we then used Mendelian randomisation to identify causal links between BMFF and osteoporosis, fracture, and T2D [11].

**Table 1:** **Comparison of DL models used for large-scale BMA analysis in UK Biobank.** Data are from the indicated studies [10, 11, 12, 13, 14, 15, 16, 38]; Xu et al. 2025a = [10]; Xu et al. 2025b = [11]. ^a^Dual-echo data (neck to knees) are from UKB category 105; multi-echo 2D data are from UKB category 126; and T1-weighted skull MRI from UKB category 100. ^b^Sample sizes are the numbers of individuals with BMA measurements after sample quality control (QC). All studies included both males and females (see original studies for further details). ^c^Exact ID of individual vertebra not specified (likely varies between participants). ^d^Note that Kaufmann et al. did not use a U-Net to segment ROIs but instead used an ANN to predict BM location and intensity; the values shown are for the overlap between true BM location vs. predicted BM location from their ANN. PCA = Principal component analysis

	Study					
Feature	Morris et al. (2024)	Xu et al. (2025a)	Xu et al. (2025b)	Wu et al. (2025)	Parkinson et al., Sorokin et al., & Ahmed et al. (2025)	Kaufmann et al. (2024)
Modality^a^	Dual-echo 3D MRI				Dual-echo 3D MRI + multi-echo 2D MRI	T1-weighted 3D MRI of the skull
**BMA measurement(s)**	BMFF of spine (*6–7 vertebrae*,* from Th8 to L3*), femoral head, total hip, and diaphysis (*all from left femur*)			BMFF of vertebrae (L1-Th8; individually and as average of L or Th groups) and left + right proximal femur	BMFF for whole spine (*Th1 to S1*), thigh and pelvis. PDFF for individual vertebra^c^	Calvarial BMA(semi-quantitative estimates)
DL model	Lightweight CBAM ROI-attention U-net (4-levels). Trained with Dice loss + Adam optimiser			Conventional 3D U-Net (“IBAS-FFCS”) with 4 levels. Trained with Dice loss + Adam optimiser	3D U-Net (further details not provided)	Artificial Neural Network (ANN) to predict location of calvarial BM cavity. Trained with loss function + Adam optimiser
Dataset for DL training & validation	Manual annotations from 75 UKB subjects (all four sites; 61–64 for training; 10–12 for validation)			Manual annotations from > 256 subjects in UKB and 180 subjects in separate cohort.	Manual annotations (dual-echo data) from 110–120 UKB subjects	Simulated dataset (336 different head types). Further validation on data from monozygotic twins.
Segmentation accuracy	Dice scores: 0.866 (diaphysis), 0.912 (spine, total hip), 0.945 (femoral head)			Dice scores from 0.863 (Th8) to 0.935 (Left proximal femur)	Dice score of 0.83 (spine), 0.82 (thigh) and 0.91 (pelvis)	Median overlap accuracy of 0.76^d^
QC within DL model	Automatic error checking (exclusion of single-voxel outputs; fusion of discontinuous ROIs).			Not described	Not described	Filtering to exclude abnormal scans
Other QC methods	Excluded extreme small or large outputs. Manually checked ROIs in training-validation dataset. PCA for QC in larger dataset (> 43k individuals)			Excluded individuals with extreme BMFF outliers (3 x IQR)	Excluded small segmentations and subjects whose 3D segmentations did not intersect with multi-echo slices.	Tested consistency in repeat scans from 30 individuals. Excluded empty or small segmentations.
Explanation for erroneous DL outputs?	Yes: biological (e.g. severe scoliosis; Non-Hodgkin Lymphoma) and technical (e.g. water-fat inversion; target ROI outside MRI volume)			Yes: biological (e.g. severe scoliosis, other abnormalities) and technical (e.g. water-fat inversion, errors in fusing MRI volumes, other image artefacts)	Not described	Yes: biological (thin calvaria) and technical (poor image quality)
Final sample size^b^	646 to 696	43,357 to 47,571	44,099 to 48,427	38,522 to 39,178	26,524 (PDFF); up to 37,589 (BMFF)	33,042
Distinct features	Efficient U-Net with attention + ROI-localisation mechanism to improve segmentation of small targets.			Fusion of separate MRI volumes allows segmentation of targets falling between each volume	Segmentations mapped to 2D multi-echo slices for PDFF measurement	Validation with simulated data; Computationally efficient.

These findings are further supported by recent work from Wu et al., who pursued similar analyses of the dual-echo UKB MRI data. Their model, a conventional 3D U-Net, was trained to segment ten individual vertebrae (Th8 to Th12 and L1-L5) and both the left and right proximal femur (LPF and RPF), allowing BMFF measurements at 12 distinct skeletal sites. They then calculated the average BMFF separately across all thoracic vertebrae, all lumbar vertebrae, and both LPF + RPF, giving a total of 15 different, site-specific BMFF measurements (Table 1). These data were used for GWAS, identification of clinical and anthropometric associates, and Mendelian randomisation to explore causal relationships [12].

The dual-echo sequences analysed by us and Wu et al. do not allow precise T2* correction to be applied to the fat fraction data, preventing measurement of the adjusted PDFF [8]. However, UKB has also conducted single-slice MRI of the pancreas and liver using multi-echo sequences, which do allow PDFF measurement [39]. These 2D slices also transect the spine, in some cases through the vertebral BM. Three recent studies took advantage of this to calculate the vertebral BM PDFF [14, 15, 16]. To do so they first trained a 3D U-Net model to segment vertebral BM, using the same dual-echo MRI data used by us and Wu et al. This gave vertebral BM segmentations from the first thoracic vertebra (T1) to the first sacral vertebra (S1). They next used this to segment vertebral BM from ~ 44,000 participants, allowing calculation of the dual-echo BMFF. Notably, they then projected these segmentations onto the single-slice liver multi-echo data, thereby defining a 2D ROI for extracting median vertebral BM PDFF. After excluding participants with missing or non-intersecting slices and those with very small segmentations, they were able to calculate vertebral PDFF data from 26,524 UKB participants [14, 16] (Table 1). Their latest study also reports BMFF measurements from the thigh and pelvis [15]. Together, these analyses allowed investigation of anthropometric variables, genetic variants, and specific diseases associated with BMFF of the thigh, pelvis and vertebrae, and vertebral PDFF.

The final UKB BMA study, from Kaufmann et al., was published before the above papers and stands out in several ways. Rather than relying on U-Net-based segmentation, they developed a custom-designed artificial neural network (ANN) to localise and estimate BMA from T1-weighted MRI scans of the skull [13]. Their ANN does so by processing one-dimensional intensity arrays extracted from the T1-weighted MRI data and interpreting the averaged signal intensity as a proxy for calvarial BMA. This allowed BMA to be estimated in over 33,000 UKB participants, followed by GWAS and analysis of associations between calvarial BMA, sex, age, and brain and body traits [13] (Table 1).

Further comparison of this ANN vs. the U-Net models is provided in later in this review and in Table 1. However, a notable limitation of the Kaufmann study is that it had access only to T1-weighted data, which cannot chemically separate the fat and water signals: other tissues can also give strong signal in T1-weighted images, making it impossible to definitively distinguish fat based on T1-weighted signal intensity alone [40]. Moreover, the relationship between fat concentration and T1-weighted signal intensity is not linear; factors like T1-bias and signal saturation can cause the signal to plateau or become non-representative of the true fat fraction [40]. Thus, Kaufmann et al. were unable to calculate the precise BMFF, making their resulting BMA estimates qualitative or semi-quantitative at best. However, previous studies have demonstrated close correlations between T1-estimated BMAT volume and dual-echo-derived BMFF [41]. Moreover, their calvarial BMA measurements show various anthropometric, genetic, and clinical associations similar to those of the BMFF/PDFF measurements reported in the studies above (Tables 2, 3 and 4). This supports their reliability as semi-quantitative BMA estimates.

**Table 2:** **Comparison of associations between BMA and anthropometric variables.** Data are from the indicated studies [10, 12, 13, 14, 15, 16]. Xu et al. 2025a = [10]. ^a^Note that Ahmed et al. did not test if thigh or pelvic BMFF is associated with BMD or BMI, or state if these differ between the sexes

	Study			
Variable	Xu et al. (2025a)	Wu et al. (2025)	Parkinson et al., Sorokin et al., & Ahmed et al. (2025)	Kaufmann et al. (2024)
BMD	BMFF at each site negatively associated with whole-body and site-specific BMD	Vertebral & femoral BMFF negatively associated with BMD	Vertebral BMFF/PDFF negatively associated with lumbar BMD^a^	Calvarial BMA negatively associated with calvarial BMD
Age	BMFF at each site increases with age	BMFF at each site increases with age	BMFF at each site, and vertebral PDFF, increase with age	Calvarial BMA increases with age in females but not males
Sex	Age-dependent: femoral BMFF higher in males at all ages; spine BMFF higher in females > 50 years	Males have lower vertebral BMFF but higher femoral BMFF vs. females	Vertebral BMFF/PDFF higher in females than males^a^	Age-dependent sex differences: calvarial BMA lower in females vs. males < 70 years but similar thereafter. Rapid increase post-menopause.
BMI	BMI correlates negatively with femoral BMFF but positively with vertebral BMFF	BMI correlates negatively with femoral BMFF but positively with vertebral BMFF	Vertebral BMFF/PDFF correlates positively with BMI in both sexes^a^	BMI correlates negatively with calvarial BMA
Peripheral adiposity	Total body fat associates positively with spine, femoral head or total hip BMFF, but negatively with diaphysis BMFF. Visceral adiposity has strong + ve association with spine BMFF, weaker + ve association with femoral head BMFF, and -ve associations with total hip and diaphysis BMFF. Associations with other adiposity traits are also site- & sex-specific	Total body fat positively correlated with BMFF at all sites; stronger association for vertebral vs. femoral BMFF	Vertebral BMFF or PDFF positively associated with visceral adiposity. Negative associations for thigh BMFF; none for pelvic BMFF. Extensive further analyses reported in Sorokin et al.	*Not assessed*
Ethnicity	Higher spine & lower diaphysis BMFF in white vs. non-white participants; further site-specific differences between white, Asian, Black, & mixed-ethnicity groups	Individuals of white ancestry have higher BMFF vs. other ancestries	*Not assessed*	*Not assessed*

**Table 3:** **Clinical implications of BMA identified from large-scale analyses in UK Biobank.** Data are from the indicated studies [11, 12, 13, 14, 15, 16]. Xu et al. 2025b = [11]

	Xu et al. (2025b)	Wu et al. (2025)	Parkinson et al., Sorokin et al., Ahmed et al. (2025)	Kaufmann et al. (2024)
Methods used	Observational (Obs) PheWAS; PRS-PheWAS; PRS predictive performance; Mendelian randomisation	Focus on genetic associations between BMFF and disease outcomes, including PRS associations, predictive performance, & Mendelian randomisation.	Targeted association studies (inc. genetic associations); case/control analyses; Mendelian randomisation.	Cases/control analysis (*osteoporosis)* & genetic correlations with specific diseases
BMA-associated diseases	For all 4 BMFF sites, Obs-PheWAS identifies associations with 47 diseases across 12 disease categories *(incident disease*) and 172 diseases across 16 categories (*incident + prevalent diseases*)	*Direct associations between BMFF and clinical diagnoses were not assessed*	Sex-specific positive associations with osteoporosis and T2D	*Direct associations between BMA and clinical diagnoses were not assessed*
Polygenic risk scores	PRSs constructed for BMFF at all four sites. PRS-PheWAS identifies associations with 22 diseases across 6 disease categories; musculoskeletal diseases (osteoporosis, fracture) are prominent.	PRS constructed for BMFF at all 15 sites. Tested relationships with 10 diseases: significance only for femoral PRSs vs. osteoporosis; no significance for fracture, T2D, or other diseases.	*Not generated*	*Not generated*
Osteoporosis	Positive associations with BMFF and PRSs for all sites. For each site, PRSs alone show moderate/good predictive power. Mendelian randomisation shows positive causal associations for BMFF at total hip and diaphysis, but not for spine BMFF.	Positive associations with femur PRSs. Poor predictive power of PRSs alone, but they improve prediction alongside other risk factors. Mendelian randomisation shows + ve associations for femoral BMFF but not vertebral BMFF.	Vertebral BMFF & PDFF higher in cases vs. controls. Mendelian randomisation shows + ve causal association for thigh BMFF only.	Calvarial BMA higher in osteoporosis patients vs. controls
Fracture	Several fracture types associate positively with directly measured BMFF or PRSs. Mendelian randomisation finds positive causal associations for femoral BMFF but only using more-lenient thresholds.	No significant relationships with PRSs for any of the 15 BMFF sites. *Mendelian randomisation not performed.*	*Not assessed*	*Not assessed*
T2D	Associates positively with spine BMFF but negatively with femoral BMFF; this persists even when controlling for peripheral adiposity. Mendelian randomisation finds significant positive causal associations for spine BMFF and negative causality for total hip and diaphysis BMFF, but only using more-lenient thresholds.	No significant relationships with PRSs for any of the 15 BMFF sites. *Mendelian randomisation not performed.*	Vertebral BMFF or PDFF + ve associated with T2D. PDFF not associated with different genetic subtypes of T2D. No causality from Mendelian randomisation.	No significant genetic correlation. Relationship with directly diagnosed T2D was not assessed.
Other notable findings	Osteoarthritis associates positively with spine BMFF & negatively with proximal femoral BMFF. Many other associations between BMFF and cardiometabolic, haematological, and oncological diseases.	Reverse Mendelian randomisation suggests that high BMD lowers BMFF	Mendelian randomisation finds -ve causal association for thigh BMFF with osteoarthritis.	Significant genetic correlations between BMA, cognitive ability & Parkinson’s disease.
Distinct features	PheWAS systematically identifies BMFF-associated diseases, shows site-specific clinical implications and identifies diseases never previously linked to BMA.	Strong focus on genetically predicted relationships between BMFF and disease, including PRSs as predictive biomarkers.	BMA analyses combined with many other imaging-derived phenotypes.	Suggests interplay between neuro-degenerative risk and calvarial BMA

Below we discuss the breakthroughs resulting from these large-scale DL-based studies, including for understanding of fundamental BMAT biology and translational insights for new clinical opportunities.

## Breakthrough 1: Insights into the Relationships Between BMA and Anthropometric Variables

Each study explored the associations between altered BMA and anthropometric traits including bone mineral density (BMD), age, sex, BMI, and body composition. The most-consistent finding is that, regardless of skeletal site or MRI method, BMA is negatively associated with BMD (Table 2). BMA also consistently increases with age, except for calvarial BMA in males [13]. Intriguingly, our study further reveals age-dependent sex differences in BMFF: males have higher femoral BMFF at all ages and higher spine BMFF when aged 40–50, but above 50 years spine BMFF becomes higher in females than males [10]. Kaufmann et al. make a similar finding for calvarial BMA, which increases rapidly post-menopause; compellingly, this increase is blunted in women taking hormone-replacement therapy [13]. Together, these findings extend smaller-scale reports of oestrogen’s ability to suppress BMAT accumulation [42].

Another common finding relates to the associations with BMI and body composition. The former relationship is positive for vertebral BMFF but negative for femoral BMFF and calvarial BMA (Table 2). Moreover, our study, Wu et al., and Parkinson et al. show that vertebral BMFF associates positively with all indices of peripheral adiposity, including visceral adipose tissue volume and total body fat % (Table 2). In contrast, Sorokin et al. and Ahmed et al. found that vertebral PDFF has only moderate, incomplete correlations with 19 other imaging-derived phenotypes (IDPs), such as liver PDFF and muscle indices; this implies that vertebral PDFF captures information that is partially distinct to that of other fat or muscle measures [15, 16]. Ahmed et al. further reveal that pelvic BMFF is not associated with peripheral adiposity at any site [15]. Regarding the proximal femur, we and Wu et al. report positive correlations with total body fat %; however, the associations between femoral BMFF and other adiposity traits are more complex, often differing between the sexes [10]. In contrast, Ahmed et al. report that thigh BMFF is negatively associated with all other peripheral adiposity traits measured but did not explore sex differences in these associations [15]. Together, these site-specific associations suggest that vertebral and femoral BMA differentially impact cardiometabolic health. Intriguingly, Ahmed et al. also find pelvic, thigh, and vertebral BMA to be positively associated with adult height [15]. However, the relationships between calvarial BMA and peripheral adiposity remain untested [13] (Table 2).

Finally, the studies from us and Wu et al. reveal site-specific differences in BMFF between white and non-white participants, with our study further investigating differences among Asian, Black, and non-white mixed-ethnicity participants (Table 2). These observations vastly extend previous reports suggesting ethnic differences in BMA [1].

Several features of these findings also give confidence in the DL-derived BMA measurements. Firstly, the vertebral PDFF data from Parkinson et al. show the same anthropometric associations as vertebral BMFF, both within their own study and when compared with data from us and Wu et al. (Table 2). This is important because it demonstrates that large-scale dual-echo BMFF measurements give similar results to multi-echo PDFF data. Secondly, Wu et al. show very similar bilateral associations for LPF and RPF, demonstrating internal consistency in their pipeline. Finally, the above associations for vertebral and femoral BMFF are generally consistent with those from previous smaller-scale studies, supporting the reliability of the large-scale BMA measurements. However, these UKB analyses are not merely replication studies: their unprecedented scale sets a new benchmark in our understanding of BMA.

## Breakthrough 2: Establishing the Genetic Architecture of BMA

**Fig. 1:**
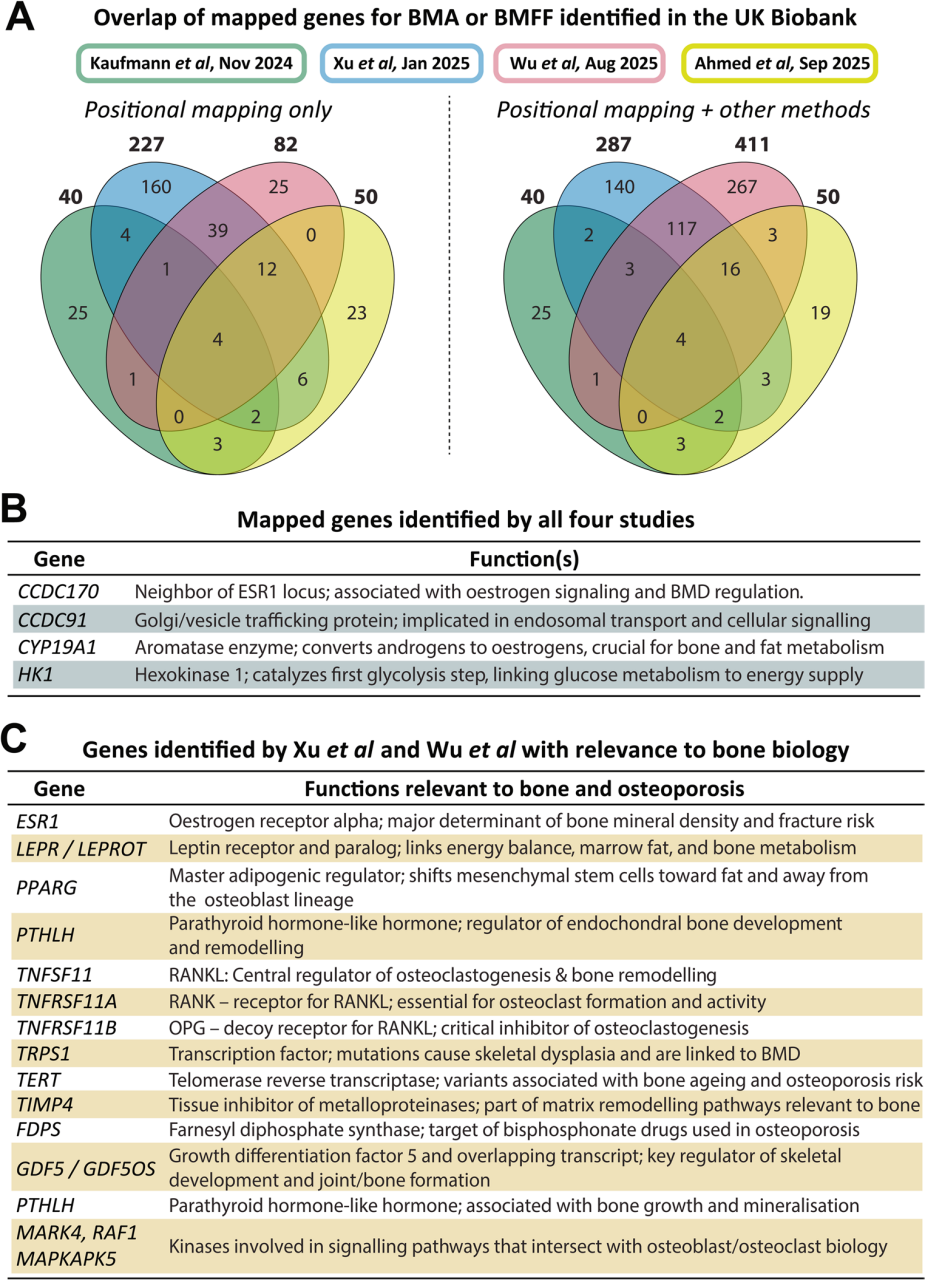
**Comparison of BMA/BMFF-associated genes identified from large-scale analyses in the UK Biobank.** (A) Genes from positional mapping are reported in Table S5 from Kaufmann et al. [13], Supplementary Data 13 from Xu et al. [10], Supplementary Data 8 of Wu et al. [12], and Supplementary Figs. 4 H-J of Ahmed et al. [15]. Those from ‘other methods’ are reported in Supplementary Data 26–29 (TWAS) from Xu et al. and Supplementary Data 19 (eQTL mapping, chromatin mapping, TWAS) from Wu et al. The total number of genes for each study are shown in bold text outside each ellipse. Note that Kaufmann et al. reported 41 independent genomic loci but two for the same gene; hence, 40 genes were used for the comparisons shown here. Ahmed et al. mention only 46 mapped genes in the text of the Results but show 49 distinct mapped genes in Supplementary Figs. 4H-J; however, these 49 genes include one locus mapping to the *NPHP3* and *ACAD11* genes. To facilitate comparison across studies, both *NPHP3 and ACAD11* were included separately, giving 50 mapped genes in total for Ahmed et al. Genes in (B-C) are those identified by positional mapping and other methods. The code and data used for these analyses is available at https://github.com/WillCawthorn/BMA_GWAS/

We, Kaufmann et al., Wu et al. and Ahmed et al. each conducted genome-wide association studies (GWAS) to identify the genetic variants associated with calvarial BMA, BMFF at each site, or vertebral PDFF [10, 12, 13, 15]. **These are the first GWASes for BMA** (Table 4; Fig. 1).

**Table 4:** **Comparison of GWAS methods and results across each study**. Other details of GWAS methods are as follows: Kaufmann et al. [13] used calvarial BMA as the exposure, adjusting for age at imaging, scanning site, and population structure of the first 20 principal components (PCs 1–20). Xu et al. and Wu et al. [10, 12] used rank-normalised BMFF as the exposure, adjusting for age at imaging visit, sex, and BMI at imaging visit; Xu et al. further adjusted for genotyping batch and PCs 1–40, while Wu et al. adjusted for PCs 1–10 (but used BOLT-LMM for GWAS). Kaufmann et al. and Xu et al. defined genome-wide significant SNPs (*P* < 5 × 10^− 8^) in low LD with each other (r^2^ < 0.6) as independent significant SNPs, whereas Wu et al. used stricter thresholds (*P* < 5 × 10^− 9^ and r^2^ < 0.1). ^a^Ahmed et al. [15] report these numbers in supplementary Figs. 4 H-J but mention only 20 (pelvic BMFF) or 21 (thigh BMFF) significant associations in their main text; details of independent SNPs and lead SNPs were not reported

		Kaufmann et al. (2024)	Xu et al. (2025a)	Wu et al. (2025)	Ahmed et al. (2025)
Multi-ancestry	**GWAS sample (n)**	*Not done (GWAS in white or non-white populations only)*	43,357 (*diaphysis*) to 47,571 (*spine*)	*Not done (GWAS in white population only)*	*Not done (GWAS in white population only)*
	**Significant associations**		121, 314, 234, & 310 independent SNPs mapping to 65, 98, 63, & 121 genes for femoral head, total hip, femoral diaphysis, & spine, respectively		
White	**GWAS sample (n)**	33,026	37,513 (*diaphysis*) to 41,024 *(spine*)	38,522 (*Proximal Femur)* to 39,178 (*Th11*)	*Not reported*
	**Significant associations**	168 independent SNPs mapping to 41 loci (40 separate genes) for calvarial BMA	67, 147, 134, & 174 independent SNPs mapping to 54, 90, 43, & 100 genes for the femoral head, total hip, femoral diaphysis, and spine, respectively	37, 32, & 31 independent SNPs mapping to 35, 30, & 25 genes for BMFF across thoracic vertebrae, lumbar vertebrae, and proximal femur.	27, 27, and 5 mapped genes for pelvic BMFF, thigh BMFF, and vertebral PDFF, respectively^a^.
	**Heritability (h** ^**2**^_**SNP**_ **)**	31.5%	Ranged from 20% (*femoral head*) to 27.5% *(diaphysis*)	Ranged from 16% (*L5*) to 23% (*Thoracic vertebrae)*	*Not reported*
	**Sex differences**	Larger effect sizes in males than females	No significant sex effects for lead SNPs (Sex x Genotype interaction), but sex-stratified meta-GWASes identify 3 sex-specific genes.	No sex effects for lead SNPs (Sex x Genotype interaction). Gene mapping identifies 33 *(male)* & 42 *(female)* sex-specific genes.	Sex-stratified GWAS identify 3 sex-specific genes. Sex x Genotype interaction not tested.
	**Other stringency analyses**	*None reported*	Meta-GWAS of two batches of BMFF data. Tested different significance thresholds for functional annotation, and GWASes for BMFF +/- BMI adjustment.	Pseudoreplication by randomly dividing into two subsets (7:3 ratio; sex and age matched); GWASes for BMFF +/- BMI adjustment	*None reported*
	**Cross-trait LDSC for genetic correlation**	Investigated 13 traits: significant negative genetic correlation with BMD and BMI	Investigate 11 traits: significant -ve genetic correlation between: (i) BMD and BMFF in all four bone regions; (ii) BMI & BMFF at each femoral site; (iii) no correlation with osteoarthritis or cardio-metabolic diseases	Investigate 23 traits: all BMFF sites show negative genetic correlation with BMD & positive correlation with osteoporosis & fracture; other relationships more site-specific.	Investigate 9 anthropometric traits. Thigh BMFF negatively correlated with BMI, WHR, and body fat %.
Non-white	**GWAS sample (n)**	4,958	5,844 (*diaphysis*) to 6,367 (*spine*)	*Not done*	*Not done*
	**Significant associations**	1 independent SNP mapping to *WNT16*	3 independent SNPs & 2 genes (femoral head); 3 independent SNPs & 1 gene (*diaphysis*). None for total hip or spine.		
	**Post-GWAS**	Analysed mapped genes’ expression in mesenchymal cells of mouse BM.	TWAS & colocalization in white population; Gene-to-function (MAGMA gene-set & tissue expression analyses) in white & multi-ancestry meta-GWASes	TWAS, colocalization, and MAGMA gene-set enrichment in white population.	MAGMA gene-set enrichment; protein QTLs to assess effects of SNPs on plasma proteins
	**Strengths**	Only GWAS for calvarial BMA	Largest sample size; the only GWAS for diaphysis BMFF and only multi-ancestry GWAS; extensive stringency tests.	Analysed individual vertebrae; combine mapping methods to increase gene discovery; extensive analysis of genetic correlations with other traits.	The only GWAS for pelvic BMFF and vertebral PDFF

The Kaufmann GWAS was the first to be publicly available, with a discovery sample of 33,042 white participants and analysis of one skeletal site (calvarial BMA). This identified significant associations with 168 independent single nucleotide polymorphisms (SNPs) that positionally mapped to 40 distinct genes (Table 4; Fig. 1A). To validate these findings, they also conducted a replication GWAS in 4,958 non-white males and females; although this identified only one genome-wide significant SNP (mapping to *WNT16*), ~ 93% of significant SNPs from the discovery GWAS showed the same effect direction in the replication GWAS [13]. Multi-ancestry GWAS was not conducted but they did report some sex differences, with larger effect sizes in males than females (Table 4).

Our study is the most comprehensive GWAS, with separate analyses in white participants, non-white participants, and a multi-ancestry analysis. Each was done in two batches, allowing meta-GWAS for increased robustness (Table 4). Across all four skeletal sites, meta-GWAS in the white population identified significant associations with 476 SNPs that, using FUMA (functional mapping and annotation), mapped to 227 genes (Fig. 1A, *left*). Spine BMFF has the most associations for any single site, with 174 independent SNPs and 100 mapped genes for participants of White ancestry (Table 4). Our multi-ancestry meta-GWAS identified even more genetic associations, with up to 121 mapped genes per site (Table 4). UKB participants are predominantly of white ancestry; hence, we focussed our further analyses within the white population, including transcriptome-wide association studies (TWAS) and colocalisation analyses. Here, the combination of GWAS mapped genes and TWAS results identified 287 BMFF-associated genes across all four sites (Fig. 1A, *right*). Sex-stratified GWASes further identified a handful of genes specific to males or females, but analysis of sex x genotype interactions revealed no overall sex differences in the genetic architecture of BMFF. In the white meta-GWAS we also tested more-stringent thresholds for functional annotation and assessed BMFF with and without BMI adjustment; in each case we obtained similar results, demonstrating the robustness of our findings.

The more-recent study from Wu et al. focussed only on the white population, conducting separate GWASes for each of the 15 BMFF sites [12]. Together, these identified a total of 145 independent SNPs that positionally mapped to 82 genes (Fig. 1A, *left*). GWASes for average BMFF across the thoracic vertebrae, lumbar vertebrae, or proximal femur identified 31–37 SNPs and 25–32 genes – similar to the numbers for calvarial BMA but fewer than those for our spine and femoral BMFF datasets (Table 4). They found tight genetic correlations between BMFF of individual vertebrae or between left vs. right proximal femur, but weaker relationships between vertebral vs. femoral BMFF. By also performing FUMA and TWAS mapping, they identified a total of 409 BMFF-associated genes across both sexes (Fig. 1A, *right*). Applying these methods in sex-stratified analyses detected 33 genes specifically in males and 42 genes specifically in females (Table 4); however, like us, they also found no overall sex x genotype interactions (Table 4).

The most recent study, from Ahmed et al., conducted GWAS not only for pelvic BMFF, thigh BMFF, and vertebral PDFF, but also for seven other adiposity traits outside the bone marrow. They provide less detail about the GWAS findings, reporting only the mapped genes and associated SNPs rather than full details of all independent SNPs associated with each trait. There is also some discrepancy in their reporting: the main results describe significant associations with 21 SNPs and mapped genes for thigh BMFF, 20 for pelvic BMFF, and 5 for vertebral BMFF, whereas their supplementary data indicate 27 SNPs and mapped genes each for thigh and pelvis [15]; herein, we use the latter numbers for comparison with the other BMA GWAS studies (Fig. 1). A distinct feature of the Ahmed et al. study is the use of protein QTL data, which suggest that pelvic BMFF is associated with plasma FLT3 concentrations. Through sex-stratified GWASes they identify three sex-specific associations with pelvic BMFF, although overall sex x genotype interactions were not assessed.

Direct comparison of the genes identified is complicated by each study using slightly different methods. For example, Wu et al. used a more-stringent GWAS p-value (5E-9) and linkage disequilibrium (LD) clumping parameter (r^2^ < 0.1) for defining independent SNPs, which reduces their number of reported SNPs and, therefore, mapped genes. Our study and that of Wu et al. categorised white participants based on genetic ethnic grouping, whereas Ahmed et al. used self-identified ethnicity ( a less-objective measure), while the basis for ethnicity categorisation in Kaufmann et al. is not clearly described. Moreover, neither Kaufmann et al. nor Ahmed et al. used TWAS to identify further genetic associations. Despite these caveats, comparing the genes identified by these studies provides further insights (Table 4; Fig. 1). For example, comparison of the GWAS mapped genes, with or without TWAS results, identifies four genes common to all three studies, with functions in oestrogen signalling *(CYP19A1*,* CCDC170)*, glucose metabolism *(HK1)* and other cellular processes (*CCDC91)* (Figs. 1A-B). The greatest overlap occurs for us and Wu et al., with 56 GWAS-mapped genes in common (Fig. 1A, *left*). Expanding the comparison to include other gene mapping approaches identifies 140 genes shared between our study and that of Wu et al. (Fig. 1A, *right*). These genes are enriched for the RANKL/OPG axis (*TNFSF11/TNFRSF11A/TNFRSF11B*), classic oestrogen signalling (*ESR1/CCDC170/CYP19A1*), adipogenesis (*PPARG/LEPR/LEPROT*), and other processes relevant to osteoporosis and skeletal remodelling (Figs. 1B-C). These comparative genomic analyses thereby provide key insights into BMAT formation and function.

All four studies also used LD score regression (LDSC) to determine genetic correlations between BMFF and other traits, including BMI, other body composition parameters, and pathological conditions. Ahmed et al. did not assess BMD, but Kaufmann, Wu, and our study each revealed significant negative genetic correlation between BMA/BMFF and BMD in all bone regions (Table 4). We and Kaufmann et al. also found consistent negative genetic correlation with BMI, something also reported by Ahmed et al. for thigh and pelvic BMFF (Table 4). However, this was not observed for any BMFF site assessed by Wu et al. (Table 4); this may be because they analysed associations between the 15 BMFF regions and 23 outcomes in total, resulting in many multiple comparisons and thus more-stringent significance thresholds. Wu et al. and Ahmed et al. also investigated genetic correlations with haematological parameters, using either LDSC [12] or Mendelian randomisation [15]. The former revealed site-specific associations with plasma triglycerides, HDL cholesterol and different blood cell types, and consistent negative genetic correlation with haemoglobin for 14 of the BMFF sites [12]. This extends decades-old observations of an inverse relationship between BMA and haematopoiesis [1]. The latter found evidence for positive causal associations between vertebral PDFF and LDL or non-HDL cholesterol [15], suggesting that vertebral BMA can directly impact systemic cardiometabolic health.

## Breakthrough 3: Understanding of the Clinical Impact of BMA

The above anthropometric associations and genetic architecture help to inform the potential clinical impact of BMA; however, six of the large-scale UKB studies have revealed far more via direct analysis of these clinical relationships (Table 3). Their methods include case/control analyses, regression for BMA vs. specific disease outcomes, testing genetic associations between BMA and pathological traits, phenome-wide association studies (PheWAS), and assessing causal relationships through Mendelian randomisation (Table 3) [11, 12, 13, 14, 15]}.

Among these, our study is the only one to pursue PheWAS to systematically identify the diseases associated with altered BMA [11] (Table 3). Observational PheWAS (Obs-PheWAS), using measured BMFF as the exposure, reveals associations with 47 incident diseases across 12 disease categories. We also established polygenic risk scores (PRS) as genetic predictors of altered BMFF at each skeletal site. These allowed PRS-PheWAS to further explore disease associations in over 300,000 people, identifying a total of 22 diseases spanning 6 disease categories (Table 3). Our PheWASes collectively reveal that altered BMFF is associated with osteoporosis, fracture, T2D, cardiovascular diseases, cancers, and many unexpected conditions that collectively place a substantial burden on public health worldwide. Notably, increased spine BMA is always linked with greater disease risk, whereas femoral BMA often shows negative associations. Moreover, Ahmed et al. performed Mendelian randomisation for 26 diseases in total, revealing that thigh BMFF has negative causal associations with knee osteoarthritis, and testing relationships beyond musculoskeletal diseases [15]. Together, these results vastly expand the scope of BMA-associated diseases.

### Osteoporosis

The full breadth of diseases is too extensive to discuss here in full. However, across all the UKB BMA studies, the most robust finding is that BMA is increased in osteoporosis, regardless of skeletal site. Indeed, in our Obs-PheWASes, osteoporosis is the only incident disease significantly associated with BMFF at all four sites, with each showing a positive association [11]. This also occurs for our PRS-PheWAS, which includes both incident and prevalent cases (Table 3). Supporting this, targeted case/control analyses from Kaufmann et al. and Parkinson et al. show that calvarial BMA and vertebral BMFF/PDFF, respectively, are increased in osteoporotic patients vs. controls [13, 14]. Wu et al. did not investigate disease associations with directly measured BMFF but instead focused on establishing PRSs and genetic correlations; the latter revealed positive relationships with osteoporosis for all 15 BMFF sites (Table 4).

We and Wu et al. also tested if our BMFF PRSs can predict osteoporosis risk. Using Cox proportional hazard models, Wu et al. identified positive associations between the proximal femoral PRSs and osteoporosis incidence, but no relationships with vertebral BMFF PRSs [12] (Table 3). Further analysis showed that their proximal femoral PRSs alone had limited predictive power for osteoporosis (C-statistics of ~ 0.53) but modestly improved predictive performance when combined with other clinical risk factors (Table 3). In contrast, each of our PRSs showed better discrimination of individuals with vs. without osteoporosis (C-statistics of ~ 0.71), even without including other risk factors [11]. This stronger predictive performance, including for spine BMFF, may result from our GWASes identifying many more genetic associations within each site, potentially allowing stronger PRSs. Whatever the explanation, the observations from us and Wu et al. support the potential of BMFF for predicting osteoporosis risk.

Notably, we, Wu et al. and Ahmed et al. used Mendelian randomisation to explore causal relationships between genetically predicted BMFF and osteoporosis. This identified causal associations with BMFF of the whole proximal femur [12], total hip and femoral diaphysis [11], or thigh BMFF [15], but not with spine/vertebral BMFF, PDFF, or pelvic BMFF (Table 3). **This is the first evidence that increased BMFF directly contributes to osteoporosis in humans.**

### Fracture

The relationships with fracture are less consistent: Kaufmann et al., Parkinson et al. and Ahmed et al. did not investigate these and, although Wu et al. identified positive genetic correlations with fracture for all 15 BMFF sites (Table 4), they found no associations with their BMFF PRSs (Table 3). Our Obs-PheWAS also found no associations with incident fractures, but we show that this likely reflects low fracture incidence in the relatively healthy UKB population [43]. Indeed, when we included prevalent cases through PRS-PheWAS or Obs-PheWAS sensitivity analysis, significant associations were detected between BMFF and various fracture subtypes [11] (Table 3).

Ours is the only study to further explore the BMFF-fracture relationship using Mendelian randomisation. As for osteoporosis, our results demonstrate evidence of causal associations that are stronger for each femoral site than for spine BMFF. However, unlike for osteoporosis, causality was not observed using more-stringent Mendelian randomisation criteria (Table 3). Future studies will therefore be important to better establish if increased BMA directly contributes to fractures.

### Type 2 Diabetes

In addition to musculoskeletal diseases, the relationship between BMA and T2D has attracted increasing attention [44]; hence, it is unsurprising that most of the recent UKB BMA studies further explored this relationship (Table 3). Intriguingly, we show that T2D associates positively with spine BMFF but negatively with BMFF at the femoral sites (Table 3). This echoes the associations with peripheral adiposity [10] and greatly extends similar recent findings in smaller-scale studies [45]. Consistent with this, Parkinson et al. show that vertebral BMFF/PDFF are positively associated with T2D [14], while Wu et al. demonstrate an inverse genetic correlation between T2D and proximal femoral BMFF [12] (Table 3). However, there is no genetic correlation with calvarial BMA, suggesting that BMAT within the skull has little systemic metabolic impact [13].

The strength of the T2D associations is further demonstrated by our sensitivity Obs-PheWAS, in which T2D is the most-significant association for femoral head, total hip and spine, and the second most-significant for diaphysis BMFF [11]. In contrast, T2D shows no associations with PRSs for BMFF, whether in our PRS-PheWASes or through the targeted analyses from Wu et al. (Table 3).

A different approach was taken by Sorokin et al., who analysed the relationship between IDPs – including vertebral PDFF – and different genetic subtypes of T2D [46]. They show that vertebral PDFF is not significantly associated with any of these subtypes, whereas other adiposity and muscle indices show strong correlations with subtypes for insulin resistance. This indicates that vertebral PDFF is a distinct axis of body composition compared to other IDPs that are classically related to insulin resistance. Consistent with this, we found that spine BMFF remains significantly associated with T2D even after controlling for variation in visceral adipose tissue or overall peripheral adiposity, highlighting independent roles for BMAT in T2D [11].

As for osteoporosis and fracture, we further explored the BMFF-T2D relationship by Mendelian randomisation. Our findings suggest that genetic predisposition to increased BMFF at the spine, total hip, and diaphysis has direct causal associations with T2D that mirror the site-specific PheWAS relationships. However, as for fracture, causality was not observed when using more-stringent Mendelian randomisation criteria (Table 3). This is supported by the Mendelian randomisation results from Ahmed et al., which find no significant causal relationships for pelvic, thigh, or vertebral BMA and T2D. These findings underscore the need for further, larger-scale BMA analyses.

## Comparison of DL Methods for BMA Analysis

It is also helpful to compare the DL methods upon which the above breakthroughs are based (Table 1). The DL pipeline from Wu et al. shares many similarities with ours, including a U-net-style encoder–decoder to segment BM regions from dual-echo 3D MRI data, with continuous optimisation using the Dice loss function and the Adam (Adaptive Moment Estimation) algorithm [47]. These segmentation masks were then applied to fat-fraction maps to quantify BMFF. Although Wu et al. used a larger training and validation cohort than us, our models achieved similar accuracies (Table 1). Parkinson et al., Sorokin et al. and Ahmed et al. briefly described their training/validation dataset and Dice scores but did not fully describe their model development (Table 1). However, Sorokin et al. state that their 3D U-Net is based on their previous models [48], suggesting similarities with the model from Wu et al.

A major advantage of our model is that it includes a convolutional layer equipped with a modified convolutional block attention module (CBAM) and an ROI-localization branch that creates an auto-zooming effect [38]. By mixing attention mechanisms with coordinate information, this substantially boosts accuracy on the femoral diaphysis – a very small, hard-to-hit target – while keeping performance competitive for the larger ROIs (spine, femoral head, and total hip). Thus, our model is advantageous for segmentation of smaller targets. In contrast, Wu et al., and presumably the Parkinson/Sorokin/Ahmed studies, used a classical 3D U-Net architecture, with default built-in parametric settings from pytorch/tensorflow packages. Compared to our attention-based model, these classical U-Nets are likely to be less computationally efficient, requiring more powerful GPUs for implementation. However, one advantage of their pipelines relates to how they processed UKB MRI data before DL segmentation. The UKBB MRI sequences consist of six volumes, with the first starting at the neck and the sixth volume extending to the knees. Our DL pipeline segments volumes 2, 4 and 5, which typically contain the spine, proximal femur (femoral head and total hip) and femoral diaphysis, respectively [38]. However, for a small proportion of participants (< 2.5%) the proximal femur and/or diaphysis fall partly or entirely outside these volumes, resulting in empty or erroneous segmentations [10, 38]. Our QC process detects and removes any faulty outputs, but this decreases the sample size. To overcome this, Wu et al. first fused the volumes into a unified 3D image using an automated fat-water swap detection and correction program, thereby preventing the problem of the segmentation target falling outside the imaging volume; a similar approach was likely taken by the Parkinson/Sorokin/Ahmed studies. Importantly, Wu et al. also confirmed that this does not compromise BMFF measurements from ROIs within the fused regions [12]. Incorporating a similar fusion, before DL segmentation, would be one way to improve our pipeline.

### Quality Control

For any large-scale application of DL, error checking is critical to ensure the reliability of the DL-derived outputs (Table 1). Our lightweight attention-based 3D U-net included dedicated error-checking steps, including automatic removal of single-voxel segmentations or segmentations with abnormal shapes (detected via deviation from the population mean), and fusion of any discontinuous ROIs [38]. Inbuilt error checking was also used by Kaufmann et al., including filters to exclude scans with abnormally high variance in skull table intensity and those with many outlier vertices; this allowed identification of scans where BM localization was likely to have failed [13] (Table 1).

No inbuilt QC is described for the models from Wu et al., Parkinson et al. or Ahmed et al., but all four models employed other QC methods, including exclusion of extreme outliers based on ROI size and/or BMFF values (Table 1). Moreover, we manually inspected all model outputs from our training and validation cohort [38], something also done by Wu et al. for a subset of their training/validation cohort. In both cases, this showed that segmentation failure could be caused by technical or biological issues (Table 1). The former include water-fat inversions, ROIs falling outside of target volumes (for our study), or failures in volume fusion (for Wu et al.). Biological issuesinclude in participants with severe scoliosis and some with non-Hodgkin lymphoma, demonstrating that rare, extreme anatomical variation can compromise DL-based BMA analyses.

Finally, an important feature of our pipeline is our use of principal component analysis (PCA) to identify anomalies among the BMFF measurements [10] (Table 1). From >46,000 participants, this identified 89 participants with very low BMFF in the femoral head and/or total hip regions, allowing manual inspection to determine if the anomalies related to technical or biological factors. A related approach is to use DL for uncertainty estimation to flag problematic segmentations or corrupted scans, reducing downstream errors in large-scale datasets [49]. We highly recommend these scalable QC approaches to others using DL to generate large-scale IDP measurements.

### How To Train your DL?

Compared to the 3D U-Net models [10, 12, 14, 38], one notable feature of the ANN from Kaufmann et al. relates to model training. Each U-Net was trained using a smaller set of MRI data that had undergone meticulous manual segmentation by experts, a common practice in medical imaging (Table 1). However, a significant challenge in medical imaging is the scarcity of high-quality, expertly annotated datasets for model training. To address this, Kaufmann et al. trained their ANN using a stimulated dataset that incorporated the natural variations in thickness and intensity of the relevant anatomical structures. This allowed creation of a massive, labelled training set without the need for laborious manual annotation. They then validated their pipeline using T1 MRI data from monozygotic twins in the Human Connectome Study, establishing high reproducibility and consistency in segmentation across genetically identical individuals. This approach of training and validation using simulated data represents a promising new paradigm for overcoming a major bottleneck in the development of medical AI applications.

Another difference is that the custom ANN from Kaufmann et al. operates on one-dimensional intensity arrays, a computationally efficient solution for localizing thin, layered structures. By contrast, 3D U-Net models process the full volumetric data, a more traditional and resource-intensive method, but one that is well-suited for segmenting complex, non-linear anatomical structures with intricate boundaries. This divergence in methodology demonstrates that, for BMA analysis and beyond, there is no single best method for DL development; the optimal approach depends on the problem and the available resources.

## Conclusions and Future Directions

DL is reshaping our understanding of BMAT and its impact on human health. Among the different methods for measuring BMA, DL pipelines for MRI analysis are by far the most mature. Histology has also benefitted from DL-powered adipocyte segmentation, providing cell-level resolution to compliment large-scale human studies. DL pipelines for ^1^H-MRS are emerging, including de-noising and QC, while those for CT-based analyses focus primarily on segmentation; however, neither modality has yet seen widespread use of DL for BMA quantification. This is an area ripe for future progress.

By using DL to measure BMA from UKB MRI data, the above studies have enabled robust, population-scale epidemiology and genetic analyses of BMA across multiple skeletal sites. The anthropometric and clinical associations are highly concordant with those from smaller-scale, manual BMA analyses, underscoring the reliability of the DL-derived BMA measurements. They also reinforce the concept that BMAT has site-specific characteristics, including for the underlying genetic architecture and for calvarial BMA, a site largely ignored by previous BMAT research.

The genetic insights highlight several challenges and opportunities. A general challenge is that UKB participants are predominantly of white European ancestry, which limits identification of BMA/BMFF-associated variants in non-white populations. Our multi-ancestry meta-GWAS provides several insights but would benefit from expanding the BMA/BMFF measurements for non-white participants. Unfortunately, other large genomic cohorts, such as the China Kadoorie Biobank [50], do not yet include population-scale MRI data, making DL-based BMA analysis impossible. Thus, a first step will be to measure BMA/BMFF across the full UKB imaging study, which recently completed MRI in all 100,000 participants. Longer-term efforts must then be made to incorporate imaging into other population-scale genomic studies.

Another key goal is to understand how BMFF/BMA-associated genetic variants impact molecular and cellular biology within bone and BM. These tissues are absent in GTEx and other large databases [51]; hence, establishing musculoskeletal eQTLs remains a major challenge to overcome. A simpler next step would be to compare the GWAS results with data from BM single-cell omics analyses to identify cell types enriched for BMFF-associated genes [52, 53]. Moreover, a significant limitation shared by each of the GWAS studies is the lack of conditional analysis and fine mapping. As a result, our current understanding of causal mechanisms remains unclear. These and other approaches could help to pinpoint the cellular and molecular mechanisms underpinning BMAT formation and function.

This knowledge will be important not only for fundamental understanding; it could also allow therapeutic targeting of BMAT to improve human health. Indeed, the most-important advances from the recent UKB studies are the breakthroughs in clinical understanding. Our PheWASes show that BMA is relevant not only to musculoskeletal, metabolic, haematological and oncological conditions, but also to diverse other diseases and disease categories; this is further supported by the genetic associations identified by Wu et al. One limitation is that UKB participants are all aged over 40: the clinical relevance of BMA for younger ages remains to be established. Nevertheless, the breadth of BMA-associated diseases should encourage interest in BMAT from other biomedical fields and motivate efforts to establish DL for clinical BMA analysis. Indeed, while the published DL pipelines perform well on standardised UKB MRI datasets, it is unclear how they will perform when applied to more-variable, real-world clinical data.

Especially noteworthy is the finding that increased femoral BMFF is causally associated with osteoporosis – the first time this has been shown [11, 12, 15]. This hugely strengthens the translational case for BMFF as a risk marker and therapeutic target. Prospective studies should now test if DL-derived BMFF, or BMFF PRSs, improves fracture risk prediction, alone or in combination with existing risk prediction tools, and whether modulating BMAT (therapeutically or via lifestyle) can improve skeletal outcomes. The evidence for causality is much weaker for fracture and T2D [11] and is only beginning to be explored for other diseases [15]. Given the scope of BMFF-associated conditions identified by our PheWASes, further investigating BMAT’s direct clinical impact should be fertile ground for future exploration.

Together, these breakthroughs position DL-enabled BMA quantification as a cornerstone for future fundamental and translational advances. This will likely involve increasingly sophisticated multi-modal DL approaches and the use of emerging foundation models, allowing imaging to be integrated with other biological and clinical data. Applying these methods beyond UKB, including to real-world clinical datasets, will further accelerate discovery and bring BMA closer to clinical practice, both for musculoskeletal diseases and beyond.

## Data Availability

The code and data for the analyses presented in Figure 1 is openly available at 10.5281/zenodo.18337336.

## Abbreviations

Adam: Adaptive Moment Estimation
ANN: Artificial neural network
BM: Bone marrow
BMA: Bone marrow adiposity
BMAT: Bone marrow adipose tissue
BMD: Bone mineral density
BMFF: Bone marrow fat fraction
CBAM: Convolutional block attention module
CNN: Convolutional neural network
CT: Computed tomography
DL: Deep learning
FUMA: Functional mapping and annotation
GWAS: Genome–wide association study
HR: pQCT–High–resolution peripheral quantitative computed tomography
IDP: Imaging-derived phenotype
LD: Linkage disequilibrium
LDSC: Linkage disequilibrium score regression
LPF: Left proximal femur
MRI: Magnetic resonance imaging
MRS: Magnetic resonance spectroscopy
PDFF: Proton density fat fraction
PheWAS: Phenome–wide association study
PRS: Polygenic risk score
QC: Quality control
ROI: Region of interest
RPF: Right proximal femur
T2D: Type 2 diabetes
TWAS: Transcriptome–wide association studies
UKB: UK Biobank
